# Effects of Food Preferences and Supplement Intake During Pregnancy on the Cleft Lip and Palate Incidence: The Japan Environment and Children’s Study

**DOI:** 10.3390/nu17193175

**Published:** 2025-10-08

**Authors:** Kumiko Fujiwara, Hazuki Tamada, Hideto Imura, Taro Matsuki, Hiroo Furukawa, Nagato Natsume, Yasuyuki Yamada, Takeshi Ebara, Michihiro Kamijima, The Japan Environment and Children’s Study (JECS) Group

**Affiliations:** 1 Oral and Maxillofacial Surgery, Osaka Medical and Pharmaceutical University, 2-7 Daigaku-Machi, Takatsuki 5698686, Osaka, Japan; kumiko.fujiwara@ompu.ac.jp; 2 Cleft Lip and Palate Center, Aichi Gakuin University Dental Hospital, Division of Research and Treatment for Oral and Maxillofacial Congenital Anomalies, School of Dentistry, Aichi Gakuin University, 2-11 Suemori-dori, Chikusa-ku, Nagoya 464-8651, Aichi, Japan; hfuru@dpc.agu.ac.jp (H.F.); natsume@dpc.agu.ac.jp (N.N.); 3 Department of Occupational and Environmental Health, Graduate School of Medical Sciences, Nagoya City University, 1 Kawasumi, Mizuho-cho, Mizuho-ku, Nagoya 467-8601, Aichi, Japan; t-matsuki@hirokoku-u.ac.jp (T.M.); yayamada@juntendo.ac.jp (Y.Y.); ebara@med.uoeh-u.ac.jp (T.E.); kamijima@med.nagoya-cu.ac.jp (M.K.); 4 Faculty of Food & Health Sciences, Aichi Shukutoku University, 2-9, Katahira, Nagakute 480-1197, Aichi, Japan; 5 Oral and Maxillofacial Surgery, Nagasaki University School of Dentistry, 1-7-1 Sakamoto, Nagasaki 852-8588, Japan; h-imura@nagasaki-u.ac.jp; 6 Department of Psychology, Faculty of Health and Wellness Science, Hiroshima International University, 555-36 Kurose-Gakuendai, Higashihiroshima 739-2695, Hiroshima, Japan; 7 Department of Health Science, Faculty of Psychological and Physical Science, Aichi Gakuin University, 12 Araike, Iwasaki-cho, Nisshin 470-0195, Aichi, Japan; 8 Graduate School of Health and Sports Science, Juntendo University, 1-1 Hiraka-gakuendai, Inzai 270-1695, Chiba, Japan; 9 Department of Ergonomics, Institute of Industrial Ecological Sciences, University of Occupational and Environmental Health, 1-1 Iseigaoka, Yahatanishi-ku, Kitakyushu 807-8555, Fukuoka, Japan

**Keywords:** non-syndromic cleft lip and/or palate, food preference, supplement, JECS

## Abstract

Background: Cleft lip and/or palate (CL/P) is a high-frequency congenital disease. Besides genetic background, maternal environmental factors may be involved in its incidence. We examined the effects of unbalanced diets and the intake of dietary supplements during pregnancy on the incidence of non-syndromic CL/P (NSCLP) via a case–control study design with multiple case groups. The case group diagnosed with NSCLP included 281 patients, 217 from the Japan Environment and Children’s Study (JECS) data and 64 from the Aichi Gakuin University Hospital from 2011 to 2014. The control group comprised 87,477 (excluding cases with multiple births, chromosomal abnormalities, or complications) of the 104,062 fetal records registered in JECS. Results: The results revealed a significantly increased risk of NSCLP (aOR 2.86, 95% CI 1.63–5.00) in mothers who avoided two or more food items out of the investigated five, i.e., milk and dairy products, eggs, soy, fish, and beef. No association was identified in mothers who avoided one food. Conclusions: Providing nutritional support when multiple foods are avoided in daily food habits may be effective in reducing the occurrence of NSCLP.

## 1. Introduction

Cleft lip and/or palate (CL/P) is the second most common congenital anomaly after heart disease and is the most common anomaly in the body [1]. It occurs at a frequency of approximately one to seven per 1000 live births worldwide [2], but regional and racial differences in the frequency are noted, with a particularly high incidence in Asia [3]. The cause of CL/P has been explained by the ‘multiple factor threshold theory’ in which genetic and environmental factors mutually influence the occurrence of CL/P [4]. It has been reported that parental and environmental factors, such as maternal age [5], obesity [5], stressful events [2], family history [6], consanguineous marriage [6], household income, education, living environment [5,7,8], maternal smoking [2], alcohol consumption [2], and drugs [5,9] may significantly impact the occurrence of CL/P. As for maternal diet, studies have reported that dairy products [5,7,10,11], drinking water [12], the intake of animal liver [8], and vegetarianism [13] may affect the CL/P incidence. These studies examined the relationship between the food groups actively consumed (“what was eaten”) and disease incidence. With regard to inadequate nutritional intake, deficiencies of zinc [8,14], iron [15,16], and folic acid, a type of B vitamin, cause many congenital diseases, including CL/P [2,4,13,17,18,19,20]. Although health risks associated with inadequate intake of individual components such as vitamins and minerals have been reported, no previous studies examined the effects of certain food groups that the mothers either avoided or had low intakes of (“what they didn’t eat”). In addition to food, various supplements are available for pregnant women. According to a recent report [21], the intake of supplements, mainly folic acid, is recommended worldwide for the prevention of congenital anomalies, and it is expected that many pregnant women in Japan also take these supplements. However, studies have reported problems caused by excessive intake of supplements [22]; thus, it is necessary to examine the effects of dietary content and supplement intake on the fetus.

This study examined the effects of an unbalanced diet and the intake of dietary supplements during pregnancy on the incidence of non-syndromic CL/P (NSCLP), focusing on food item avoidance and supplement intake during pregnancy.

## 2. Materials and Methods

### 2.1. Study Design and Participants

To yield high statistical power, we adopted a case–control study design in which multiple case groups were combined, i.e., those from the Japan Environment and Children’s Study (JECS), and a survey of CL/P patients at the Aichi Gakuin University dental hospital (AGU).

The JECS protocol was reviewed and approved by the Ministry of the Environment Institutional Review Board on Epidemiological Studies and by the Ethics Committees of all participating institutions. Written informed consent was obtained from all participants. The study was conducted in accordance with the Declaration of Helsinki and other national regulations and guidelines. Pregnant women were recruited between January 2011 and March 2014 for the Japan Environment and Children Study (JECS), which is a nationwide large-scale prospective cohort study, registered in the UMIN Clinical Trials Registry (number UMIN000030786). Expectant mothers were eligible to participate in this study if they resided in the Study Areas at the time of recruitment, had their due date after 1 August 2011, and were fluent Japanese speakers who could understand and complete a set of self-administered questionnaires [23,24,25]. Participants’ medical records were transcribed by physicians, midwives/nurses, and/or Research Co-ordinators at registration, immediately after delivery, and at 1 month after delivery.

AGU is the university hospital where most CL/P patients in one of the JECS Areas (Aichi) are treated. The eligibility criteria of the participants in the retrospective survey at AGU are the same as those of the JECS. The study protocol was approved by the Ethics Committee of Aichi Gakuin University (no.327). The AGU participants were asked questions that were equivalent to those used in the JECS. Participants’ medical records were extracted and transcribed retrospectively by physicians.

**Figure 1:**
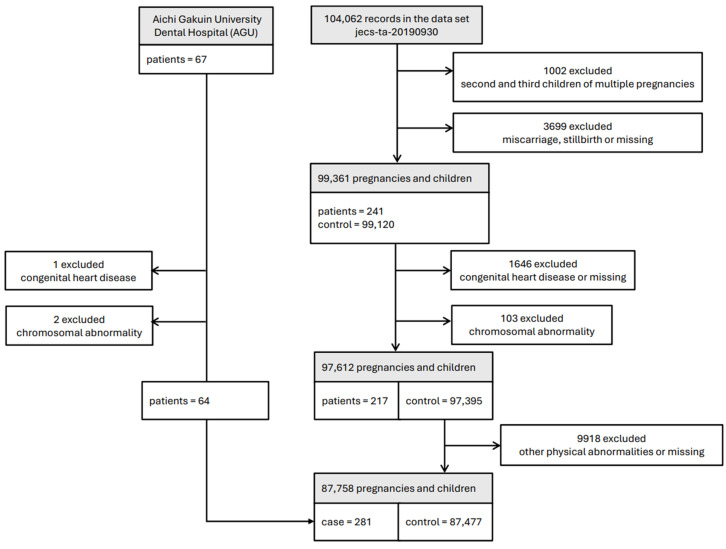
Flow diagram summarizing the study recruitment process.

Figure 1 shows a flow diagram summarizing the study recruitment process. Of the 104,062 fetal records registered in JECS (jecs-ta-20190930), participants who were repeatedly invited to the study with their second or later births (*n* = 1002), miscarriages and stillbirths (*n* = 3699), newborns with cardiac diseases (*n* = 1646), or chromosomal abnormalities (*n* = 103) were excluded (217 patients and 97,395 non-patients). From these non-patients, children with any physical abnormalities (*n* = 9918) were also excluded. Children without obvious diseases were defined as the control group (*n* = 87,477), and 217 patients with non-syndromic CL/P (NSCLP), i.e., CL/P with no complications suggestive of syndromic CL/P, were selected as patients. Moreover, 64 patients attending the AGU with NSCLP whose mothers became pregnant during almost the same years as those recorded in the JECS were included in the NSCLP group; therefore, the total number of eligible patients with NSCLP was 281.

### 2.2. Variables

Data on the socio-demographic and lifestyle characteristics in the JECS were obtained through a questionnaire survey during the second/third trimester of pregnancy. NSCLP occurrence as an outcome was transcribed from the medical records at the time of delivery and at 1 month after delivery. In the AGU, questionnaires about life at the time of pregnancy were administered during postnatal visits, and disease information was collected from patients’ medical records. Exposure factors included the pattern of foods avoided during pregnancy (milk and dairy products, eggs, soy, fish, beef and peanuts) and supplements taken (folic acid, zinc, eicosapentaenoic acid [EPA], docosahexaenoic acid [DHA], and lactobacillus beverages). To examine the association between the number of foods avoided and the outcome, the number of foods avoided was divided into three categories (0, 1, and ≥2). Covariates included maternal smoking history (no vs. yes), paternal smoking history (no vs. yes), maternal passive smoking history (no vs. yes), alcohol intake during pregnancy (no vs. yes), maternal highest level of education (junior high school/high school, technical junior college or technical/vocational college or associate degree, bachelor’s degree, graduate degree), and annual household income (<400, 400–<800, 800–<1200, ≥1200, and 1000 JPY × 10,000). When assessing the association between the number of foods avoided and the outcome, taking supplements (no vs. yes) was added as a covariate.

### 2.3. Statistical Analysis

The association of food and supplement intake with NSCLP occurrence was assessed via logistic regression analysis. Odds ratios (ORs) were adjusted for confounders as described above, and crude and adjusted ORs (aORs) and 95% confidence intervals (95% CIs) are presented.

Missing values were complemented by multiple imputations, and q-values were calculated for aORs using the Benjamini–Hochberg method (R, version 4.2.1) to correct for the false discovery rate. A q-value < 0.05 was considered statistically significant. All statistical analyses, except for the calculation of q-values, were performed using SPSS, version 23 (IBM Corp., Tokyo, Japan).

## 3. Results

The basic attributes of mothers and their lifestyle habits during pregnancy are shown in Table 1. The mean age of the mothers at enrollment was 30.63 ± 5.05 years for the JECS, and 30.38 ± 5.38 for the AGU. Mothers answered that they avoided milk and dairy products (1.8%), eggs (1.9%), soy (0.3%), fish (2.2%), beef (1.1%), and peanuts (1.9%) during pregnancy. The supplements taken during pregnancy were folic acid (41.5%), zinc (4.3%), EPA (1.4%), DHA (2.7%), and lactic acid beverages (51.1%). The patterns of foods avoided and supplements taken during pregnancy were as follows: without food avoidance and supplement intake (27.9%), with food avoidance but without supplement intake (2.0%), without food avoidance but with supplement intake (62.2%), and with food avoidance and supplement intake (4.9%).

**Figure 2:**
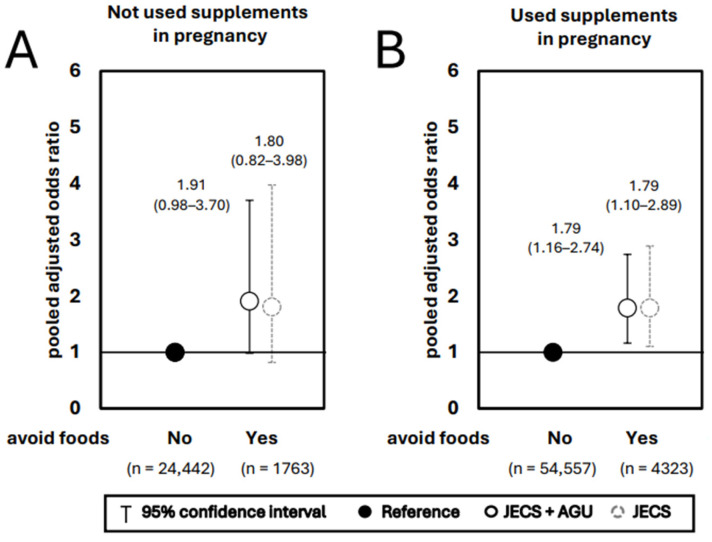
Association of avoided foods and non-syndromic cleft lip and/or palate (NSCLP) stratified by supplements intake. Adjusted for maternal smoking history, paternal smoking history, maternal passive smoking history, maternal drinking history, maternal educational status, and annual income.

A stratified analysis of the association between the pattern of presence or absence of foods avoided and supplement intake during pregnancy and NSCLP occurrence is shown in Figure 2. In the case of not taking supplements, the presence or absence of foods avoided was not significantly associated with NSCLP occurrence (Figure 2A). In the case of taking supplements, the risk of NSCLP occurrence was significantly increased for those reporting the presence of avoided foods compared to those reporting the absence of such foods (aOR 1.79 and 1.79, 95% CI 1.16–2.74 and 1.10–2.89, q = 0.03 and 0.04 for JECS + AGU data and JECS data only, respectively) (Figure 2B).

**Figure 3:**
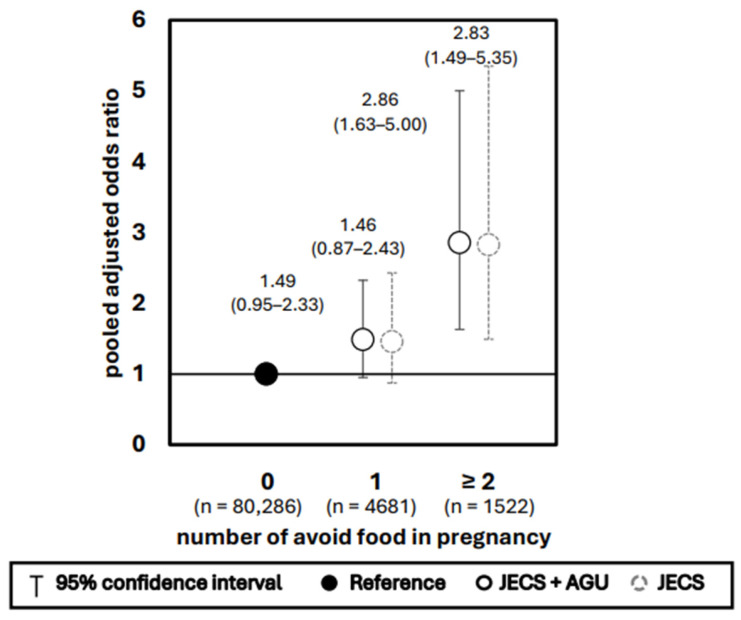
Impact of number of avoided foods in pregnancy with NSCLP. Adjusted for supplement use in pregnancy, maternal smoking history, paternal smoking history, maternal passive smoking history, maternal drinking history, maternal educational status, and annual income.

The association between the number of foods avoided and the occurrence of NSCLP is shown in Figure 3. A significantly increased risk was observed only when two or more foods were avoided during pregnancy (aOR 2.86, 95% CI 1.63–5.00, q < 0.001). In the group with one food avoided during pregnancy, the most avoided food was fish (32.4%), whereas in the group with two or more avoided food items, dairy products (24.8%) and eggs (24.4%) were the most avoided.

## 4. Discussion

### 4.1. Effects of Avoiding Specific Foods

To our knowledge, this is the first study to show that avoiding two or more of the investigated six food items during pregnancy might lead to an increased risk of NSCLP. As for fetal exposure, the effects of maternal smoking, consumption of alcohol and other tobacco products [2], drug use [5], and X-ray exposure have been reported previously [26]. Many studies have also examined possible increased/decreased risk on foods, such as dairy products [7,10,11], drinking water [12], zinc [3,8,27], and iron [16], trace elements such as selenium [14,15], and especially on foods and supplements containing folic acid [16,17,28,29]. Most previous reports relating to food intake and the occurrence of congenital anomalies have examined the food groups (“what was eaten”) that were actively consumed [7,8,10,11,12]. However, nutrient deficiencies may affect the fetus [2,4,8,13,14,15,16,17,18,19,20]; therefore, the food groups that were consumed less (“what was not eaten”) should be examined. While the effect of extreme dietary restrictions, such as vegetarian and vegan diets, has been reported, few studies have investigated the effects of avoiding food in general population. Only Sutapa et al. reported that the so-called “exclusive vegetarianism,” the absence of animal-derived foods (nutrients), can lead to an unbalanced nutritional status and can consequently cause the development of orofacial cleft [13]. Our large cohort study revealed the evidence that dietary behavior with more than one food avoidance might be a risk for NSCLP, even in the absence of extreme dietary restrictions such as vegetarian or vegan diets. Furthermore, our results showed that food avoidance significantly affected the occurrence of NSCLP only when supplements were used. Though this might have resulted from the difference in the statistical power attributable to the sample size, the results suggest that maternal nutritional deficiencies caused by such food avoidance may not have been compensated for by supplement intake.

### 4.2. Effects of Taking Supplements

Many different types of supplements are available on the market, and many products are recommended for use before and during pregnancy. Vitamin and mineral supplements prevent the occurrence of many congenital anomalies, including CLP [17,18,23,28,29], but a preventive effect of these supplements remains unclear. Regarding the intake of multivitamins, the risk of occurrence of CLP was reduced when they were taken with folic acid [30], whereas Garland et al. reported no effect [5]. In addition, fat-soluble vitamins in multivitamin preparations, especially vitamin A, are involved in teratogenicity [31] and should be taken with caution during pregnancy [1,17,32]. Excessive intake of vitamin E is also a cause of concern for negative fetal effects [22]. Previously, Sato et al. [33] reported that the use rate of supplements among pregnant women in Japan was approximately 75%, and 50% of pregnant women took folic acid and other supplements at the same time. In the present study, 67.1% of the total respondents reported taking supplements during pregnancy, thereby suggesting a good understanding of the nutrient requirements during pregnancy. Ishikawa et al. reported that 31% of pregnant Japanese women took folic acid supplements during pregnancy [34], but only 15% received sufficient amounts of folic acid (400 µg/day) [35]. In other words, although many pregnant women are aware of the importance of nutrients such as folic acid that are necessary during pregnancy and actively take supplements, they do not always receive the necessary amount. Regarding the kinds of supplements, there have been no reports on the effect of intake of Lactobacillus products as supplements on the occurrence of congenital anomalies. While our previous case–control study showed that mothers’ intake of dairy products (milk and cheese) containing lactobacilli during pregnancy in the NSCLP group was less than that in the normal babies group [11], chi-square test results in the present study without covariate adjustment (Table 1) showed that the intake of Lactobacillus products was not associated with NSCLP occurrence. Supplementation of zinc, of which the requirement of the fetus is high, thus putting the mother at risk of zinc deficiency [36,37], also had no significant association in the crude analyses. Both EPA and DHA, which are essential fatty acids not synthesized by the body, and necessary for the maintenance of the fetal brain and the development of the nervous system during fetal life [38] showed the same.

### 4.3. Limitations

This study had some limitations. First, information about exposure to foods avoided during pregnancy and supplement intake might have introduced recall biases owing to the retrospective nature of the surveys in the cases recruited in the AGU. This retrospective reporting may also have caused some misclassification of exposures, although the similar results from the JECS prospective data suggests that our main findings are reliable. Second, the variables related to being picky about foods were collected as dichotomous ones, without details on frequency, quantity, or specific period. This limitation reduced the precision of dietary assessment and prevented evaluation of possible dose–response effects. Third, we could not adjust for potential confounding factors, such as folic acid intake. Supplement and food intake were also recorded only as yes or no, without information on dosage, duration, timing, or brand. Fourth, this was an observational study; thus, we could not identify underlying mechanisms for why slight food avoidance increases NSCLP risk. Because of this design, any direct causal relationship remains speculative, and the observed associations should be interpreted with caution. Fifth, our study population consisted only of Japanese mothers, whose dietary culture, supplement habits, and genetic background may differ from those of other populations. Therefore, the generalizability of our findings to non-Japanese populations may be limited. Lastly, this study was conducted using available items of exposures commonly used for both the JECS and AGU surveys to ensure data compatibility, resulting in the available variables about participants’ dietary habits being limited. Combining JECS and AGU data may also have caused some heterogeneity. In addition, although supplements are generally considered protective, our finding of increased risk with food avoidance should be interpreted cautiously, as the mechanisms are not fully understood.

## 5. Conclusions

Our study showed a significantly increased risk of NSCLP when two or more food avoidances of the investigated five were present. Providing nutritional support when multiple foods are avoided in daily food habits may be effective in reducing the occurrence of NSCLP.

## Figures and Tables

**Table 1:** Maternal characteristics of participants.

		Total (%)		JECS					JECS and AGU		
				Control (%)		Patients (%)		*p*-Value	Patients (%)		*p*-Value
Avoid intake of milk and dairy products in pregnancy								0.29			0.17
	No	84,912	(96.8)	84,644	(98.2)	206	(97.2)		268	(97.1)	
	Yes	1578	(1.8)	1570	(1.8)	6	(2.8)		8	(2.9)	
	Missing	1268	(1.4)								
Avoid egg intake in pregnancy								1.00			1.00
	No	84,791	(96.6)	84,520	(98.0)	208	(98.1)		271	(98.2)	
	Yes	1699	(1.9)	1694	(2.0)	4	(1.9)		5	(1.8)	
	Missing	1268	(1.4)								
Avoid soy intake in pregnancy								0.51			0.60
	No	86,198	(98.2)	85,924	(99.7)	211	(99.5)		274	(99.6)	
	Yes	291	(0.3)	290	(0.3)	1	(0.5)		1	(0.4)	
	Missing	1269	(1.4)								
Avoid fish intake in pregnancy								0.00			0.00
	No	84,525	(96.3)	84,266	(97.7)	200	(94.3)		259	(93.8)	
	Yes	1965	(2.2)	1948	(2.3)	12	(5.7)		17	(6.2)	
	Missing	1268	(1.4)								
Avoid beef intake in pregnancy								0.03			0.00
	No	85,536	(97.5)	85,269	(98.9)	206	(97.2)		267	(96.7)	
	Yes	954	(1.1)	945	(1.1)	6	(2.8)		9	(3.3)	
	Missing	1268	(1.4)								
Avoid peanuts intake in pregnancy								0.02			0.05
	No	84,815	(96.6)	84,549	(98.1)	203	(95.8)		266	(96.4)	
	Yes	1675	(1.9)	1665	(1.9)	9	(4.2)		10	(3.6)	
	Missing	1268	(1.4)								
Folic acid supplements use in pregnancy								0.23			0.14
	No	49,737	(56.7)	49,594	(57.8)	112	(53.6)		143	(53.2)	
	Yes	36,391	(41.5)	36,265	(42.2)	97	(46.4)		126	(46.8)	
	Missing	1630	(1.9)								
Zinc supplements use in pregnancy								0.50			0.14
	No	82,094	(93.5)	81,843	(95.6)	197	(94.7)		251	(93.7)	
	Yes	3811	(4.3)	3794	(4.4)	11	(5.3)		17	(6.3)	
	Missing	1853	(2.1)								
EPA supplements use in pregnancy								0.77			0.60
	No	84,540	(96.3)	84,273	(98.6)	206	(99.0)		267	(99.3)	
	Yes	1233	(1.4)	1231	(1.4)	2	(1.0)		2	(0.7)	
	Missing	1985	(2.3)								
DHA supplements use in pregnancy								0.67			0.26
	No	83,458	(95.1)	83,193	(97.2)	204	(98.1)		265	(98.5)	
	Yes	2366	(2.7)	2362	(2.8)	4	(1.9)		4	(1.5)	
	Missing	1934	(2.2)								
Lactic acid drink supplements use in pregnancy								0.63			0.39
	No	41,217	(47.0)	41,081	(47.9)	96	(46.2)		136	(50.6)	
	Yes	44,813	(51.1)	44,680	(52.1)	112	(53.8)		133	(49.4)	
	Missing	1728	(2.0)								
Avoid foods and use supplements in pregnancy								0.05			0.01
	Avoid foods: No Use supplements: No	24,442	(27.9)	24,370	(28.7)	53	(25.6)		72	(27.2)	
	Avoid foods: Yes Use supplements: No	1763	(2.0)	1753	(2.1)	7	(3.4)		10	(3.8)	
	Avoid foods: No Use supplements: Yes	54,557	(62.2)	54,397	(64.1)	129	(62.3)		160	(60.4)	
	Avoid foods: Yes Use supplements: Yes	4323	(4.9)	4300	(5.1)	18	(8.7)		23	(8.7)	
	Missing	2673	(3.0)								
Maternal smoking history								0.84			0.90
	No	49,421	(56.3)	49,263	(57.6)	119	(56.9)		158	(58.1)	
	Yes	36,332	(41.4)	36,218	(42.4)	90	(43.1)		114	(41.9)	
	Missing	2005	(2.3)								
Paternal smoking history								0.70			0.37
	No	22,998	(26.3)	22,918	(27.1)	59	(28.4)		80	(29.5)	
	Yes	61,852	(70.5)	61,661	(72.9)	149	(71.6)		191	(70.5)	
	Missing	2908	(3.3)								
Maternal passive smoking								1.00			0.71
	No	53,480	(60.9)	53,313	(62.0)	131	(62.1)		167	(60.9)	
	Yes	32,750	(37.3)	32,643	(38.0)	80	(37.9)		107	(39.1)	
	Missing	1528	(1.7)								
Alcohol intake during second/third trimesters								0.89			0.16
	No	28,753	(32.8)	28,650	(33.5)	72	(34.0)		103	(37.6)	
	Yes	57,031	(65.0)	56,860	(66.5)	140	(66.0)		171	(62.4)	
	Missing	1974	(2.2)								
Maternal educational status								0.65			0.85
	Junior high school or high school	31,556	(36.0)	31,452	(36.7)	85	(40.7)		104	(38.1)	
	Higher professional school or professional school	36,183	(41.2)	36,074	(42.1)	80	(38.3)		109	(39.9)	
	Junior college or college	17,021	(19.4)	16,964	(19.8)	41	(19.6)		57	(20.9)	
	Postgraduate college	1213	(1.4)	1210	(1.4)	3	(1.4)		3	(1.1)	
	Missing	1785	(2.0)								
Annual income (JPY × 10,000)								0.34			0.07
	<400	32,528	(37.1)	32,439	(40.6)	76	(39.2)		89	(34.6)	
	400–<800	39,120	(44.6)	38,987	(48.8)	95	(49.0)		133	(51.8)	
	800–<1200	7077	(8.1)	7045	(8.8)	22	(11.3)		32	(12.5)	
	≥1200	1480	(1.7)	1477	(1.8)	1	(0.5)		3	(1.2)	
	Missing	7553	(8.6)								

JPY, Japanese yen; JECS, the Japan Environment and Children’s Study; AGU, the Aichi Gakuin University dental hospital; DHA, docosahexaenoic acid; EPA, eicosapentaenoic acid.

## Data Availability

The corresponding author had full access to all study data and took final responsibility for the decision to submit for publication. Data are unsuitable for public deposition due to ethical restrictions and legal framework of Japan. It is prohibited by the Act on the Protection of Personal Information (Act No. 57 of 30 May 2003, amendment on 9 September 2015) to publicly deposit the data containing personal information. Ethical Guidelines for Medical and Health Research Involving Human Subjects, enforced by the Japan Ministry of Education, Culture, Sports, Science and Technology and the Ministry of Health, Labour and Welfare also restricts the open sharing of the epidemiologic data. All inquiries about access to data except for those obtained in the AGU should be sent to: jecs-en@nies.go.jp. The person responsible for handling enquiries sent to this e-mail address is Dr Shoji F. Nakayama, JECS Programme Office, National Institute for Environmental Studies.
